# Exploring the Impact of Auditory Hallucinations on Sudden Sensorineural Hearing Loss in Adulthood: A Case Report

**DOI:** 10.7759/cureus.53764

**Published:** 2024-02-07

**Authors:** Camryn Daidone, Hitesh P Rai, Kimberly Loveless

**Affiliations:** 1 Research, Edward Via College of Osteopathic Medicine, Shreveport, USA; 2 Psychiatry, Brentwood Hospital, Shreveport, USA

**Keywords:** hearing impairment, clinical psychiatry, schizophrenia and other psychotic disorders, sudden sensorineural hearing loss (ssnhl), auditory hallucinations

## Abstract

Auditory hallucinations are sounds that patients perceive as coming from outside of their body. Though the mechanism causing auditory hallucinations is not entirely understood, there is a significant amount of evidence suggesting that auditory hallucinations leave lasting impacts on the brain in the same regions that are involved in auditory processing. Sudden sensorineural hearing loss (SSNHL) is a poorly understood condition in which patients lose their hearing typically in the fifth decade of life. Here we present a case of a 42-year-old female with a history of schizophrenia with auditory hallucinations who experienced SSNHL at age 40. As the patient had no known risk factors for SSNHL, we propose that this patient’s SSNHL is linked to her history of auditory hallucinations. Through the presentation of this case, we hope to explore the pathogenesis of auditory hallucinations and investigate a potentially bidirectional association between auditory hallucinations and SSNHL. This study calls for further investigation into the impacts of auditory hallucinations on the brain, possible etiologies of SSNHL, and the possibility that auditory hallucinations serve as a risk factor for SSNHL.

## Introduction

Paracusis, or auditory hallucinations, are sounds that patients perceive as coming from outside of their body but do not exist to others. They can range in frequency and intensity from occasional quiet indistinct noises to frequent distinct voices or music that can be quite disturbing [1]. It is estimated that anywhere from 5-28% of individuals experience auditory hallucinations [1]. Auditory hallucinations are classically known as symptoms of psychiatric conditions like schizophrenia and bipolar disorder; they may also exist in the context of drug use or other medical problems such as migraines and hearing loss [1].

Though the pathogenesis of auditory hallucinations is not entirely understood, current research has linked auditory hallucinations to dysfunctions in regions of the brain such as the superior and middle temporal gyri, the inferior frontal gyrus, the medial prefrontal cortex, the amygdala, and the posterior cingulate gyrus [1,2]. Research has suggested that dysfunctions in neurotransmitters such as serotonin and dopamine are integral in the pathogenesis of auditory hallucinations.

Sudden sensorineural hearing loss (SSNHL) is a poorly understood condition in which patients experience a hearing loss of 30dB or more over three frequencies within a three-day period. This condition most frequently affects adults in their 40s or 50s, and approximately 90% of cases of SSNHL are considered idiopathic [3]. Approximately 5-20/100,000 individuals develop idiopathic SSNHL each year. Though most cases of SSNHL are not linked to a particular cause, some risk factors have been identified, such as persistent viral illness, hypercoagulation disorders, and the use of ototoxic drugs including some antibiotic and chemotherapeutic agents [3,4]. Patients with SSNHL are at an increased risk of developing depressive and anxiety disorders [5,6]. Some studies indicate that there may be a bidirectional relationship between depression and sensorineural hearing loss, wherein patients with SSNHL are likely to develop depression due to increased loneliness and stress associated with their disability, while patients with depression may develop decreased global cognitive functioning that weakens auditory processing centers and may leave patients susceptible to hearing loss [7].

There is a substantial amount of research suggesting that individuals who are hearing impaired are at an increased likelihood of developing psychosis and auditory hallucinations [8-10]. Additionally, several studies suggest that patients who have schizophrenia or schizoaffective disorders with auditory hallucinations have poorer hearing than the general population [11-14]. Still, there is very little research investigating auditory hallucinations as a potential risk factor for SSNHL.

Here we present the case of a patient with a long history of schizophrenia with frequent auditory and visual hallucinations who experienced sudden sensorineural hearing loss at age 40. Through the presentation of this case, we hope to explore the pathogenesis of auditory hallucinations and investigate a potentially bidirectional association between auditory hallucinations and SSNHL.

## Case presentation

A 42-year-old African American female with a history of poorly controlled schizophrenia presented to an inpatient psychiatric institution via a physician emergency certificate after running into the street because of auditory hallucinations with voices instructing her to kill herself. The patient was diagnosed with schizophrenia at age 15 and had a long history of psychosis, visual hallucinations, and auditory hallucinations that the patient described as sounding like a “superdome full of people.” She denied symptoms consistent with depression, anxiety, or suicidal and/or homicidal ideation, except when the voices were telling her to end her life. She had several episodes of psychosis including one other significant suicide attempt and several psychiatric hospitalizations throughout her life.

Her past medical history included iron deficiency anemia, carpal tunnel syndrome, and a bilateral SSNHL that occurred less than two years prior to presentation. The patient is obese, which she reported may be a result of antipsychotic medications causing her to gain weight. The patient had no other significant past medical or surgical history.

The patient reported that her sensorineural hearing loss happened “all of a sudden” at age 40, with no previous history of hearing impairment. At the time of presentation, she was using hearing aids which provided only some improvement in her hearing. She primarily communicated through reading lips. She had previously taken multiple psychotropic medications in the past, including olanzapine, aripiprazole, risperidone, amitriptyline, benztropine, and valproic acid. She also previously took semaglutide for weight loss but had discontinued that medication. She reported an allergy to risperidone, which caused a rash and “eyes rolling back” in her head. At the time of presentation, she was taking an iron supplement and no other medications. The patient denied any substance use and a urine drug screen was negative upon admission.

The patient’s family history was significant for bipolar diagnosis in her mother and sister, both well-controlled with medication. The patient had four children under the age of 20, none of whom had any mental health diagnoses. The patient had no family history of hearing loss.

During this eight-day hospitalization, the patient was prescribed haloperidol and valproic acid, which she tolerated well and which were successful in significantly decreasing this patient’s psychosis. The patient was discharged on haloperidol and valproic acid with a referral to outpatient services. At the time of discharge, she denied any suicidal ideation, homicidal ideation, paranoia, or visual hallucinations, and reported that the “voices have quieted down.” The patient provided written consent to the use of all information in their medical record for the report of this case.

## Discussion

The present study reports the case of a patient with a long history of auditory hallucinations who experienced SSNHL at age 40. This patient had none of the known risk factors for SSNHL, including viral illness, coagulopathies, or the use of ototoxic drugs [3,4]. She had no known family history of SSNHL and had no hearing impairment prior to the sudden loss of hearing at age 40. Due to the lack of other identifiable risk factors, it is possible that this patient’s SSNHL was associated with her history of frequent vivid auditory hallucinations.

**Figure 1:**
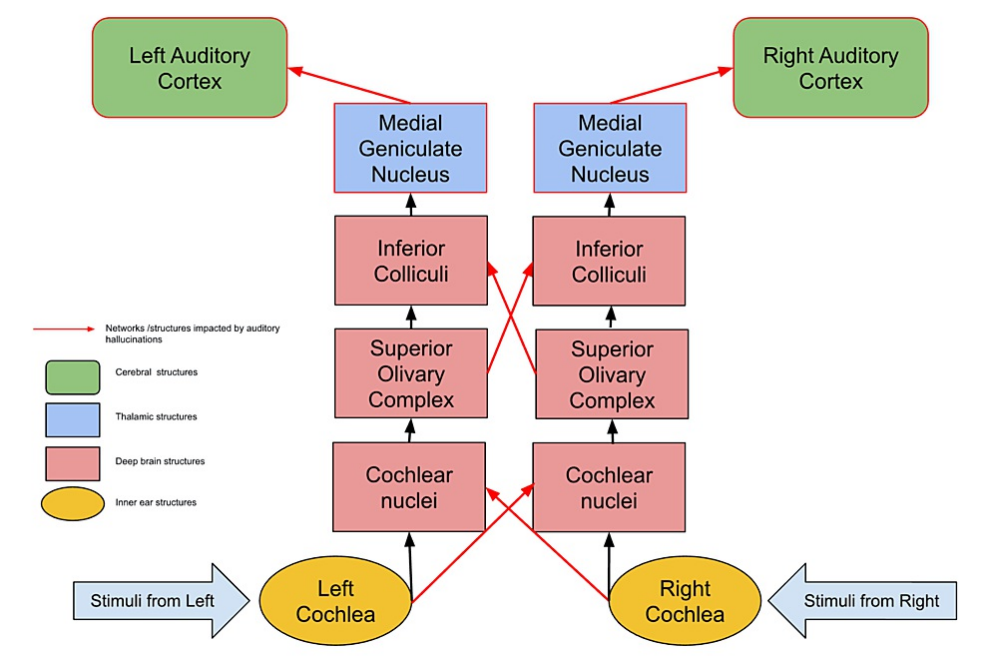
The auditory processing pathway. Structures and neural connections that are known to be impacted by auditory hallucinations are indicated in red Image Credit: Author Camryn Daidone

In the absence of disease, auditory processing follows a stereotypical system through the brain that is outlined in Figure 1. Sounds are first perceived by the cochlea and transmitted via cranial nerve eight (CN VIII) to the brainstem, where they are transmitted sequentially through the cochlear nucleus, superior olivary complex, and inferior colliculus. These signals are then processed in the thalamus at the medial geniculate nucleus and transmitted to the primary auditory cortex, which is positioned near the superior gyrus of the temporal lobe on both the ipsilateral and contralateral sides [15,16]. Throughout this process, there are several decussation sites where neural signals pass to the contralateral side of the brain. The interhemispheric processing of auditory stimuli is imperative in perceiving sounds from both sides of the body and provides a more robust network so that hearing is less likely to be fully lost in the case of brain damage [15,16].

Though the neuronal networks underlying auditory hallucinations are not entirely understood, research using functional MRI (fMRI) data has highlighted some potential impacts of auditory hallucinations on the brain. First, patients who experience frequent auditory hallucinations were shown to have stronger connections between the medial geniculate nucleus of the thalamus and the auditory cortex of the temporal lobe, and the medial geniculate nucleus may atrophy as a result. fMRI data found that the medial geniculate nucleus was smaller in patients who had a history of auditory hallucinations than those who did not [17]. Research suggests an increase in connectivity between the auditory and language cortexes of the brain and deep brain structures during auditory and visual hallucinations [18]. One study evaluating the efficacy of deep brain stimulation on auditory hallucinations showed that reprogramming of the superior temporal gyrus, a region of the primary auditory cortex, was successful in decreasing the frequency of auditory hallucinations [2].

Another study analyzing fMRI data during auditory hallucinations suggests that during the very first episode of auditory hallucination, interhemispheric connections in the auditory pathway are very strong, but over time this interhemispheric network weakens [18]. Multiple studies have also noted that patients with auditory hallucinations fail to demonstrate a right ear advantage and appropriate attention shift based on stimuli [19]. Additional studies have suggested the possibility of disproportionate impairments in the right cerebral hemisphere compared to the left [20]. These findings support the theory that auditory hallucinations lead to a weakened interhemispheric network in the auditory processing center. Figure 1 highlights the specific neural networks and structures in the auditory processing pathway that are known to be impacted by auditory hallucinations.

Additionally, these findings demonstrate a significant overlap between the known ascending path of auditory processing and the proposed impacts of auditory hallucinations on the brain. These may serve as the mechanisms behind the known decreased hearing in patients with auditory hallucinations. Here we suggest that these impacts of auditory hallucinations may have predisposed the patient in this study to develop SSNHL.

It is important to also note some other theories on the impact of auditory hallucinations on progressive hearing loss. Many researchers suggest a neurotransmitter-mediated change in auditory processing, such as dysfunctions in N-methyl-D-aspartate (NMDA), impairing perception in the auditory cortex [21]. Other theories propose that the impairment in auditory processing in individuals with auditory hallucinations may be masked by impairments in semantic processing, facial recognition, and linguistics. These deficits may not be linked directly with the processing of auditory stimuli but rather with the interpretation of language, which can be linked to impairments in the frontotemporal and parietal networks of the brain [22,23].

Studies evaluating the bidirectional relationship between depression and SSNHL proposed that a global decrease in cognitive function in patients with depression impacts auditory processing through similar mechanisms in which patients with cognitive impairment are likely to experience hearing loss [7,24]. Though auditory hallucinations, particularly those associated with schizophrenia, are typically associated with an increase in neural networks and increases in neurotransmitters such as dopamine and serotonin, chronic overuse may lead to decreased sensitivity to stimuli and mimic the decrease in cognitive function as seen in patients with depression [25].

## Conclusions

Here we present the case of a 42-year-old female with an extensive history of schizophrenia and auditory hallucinations who experienced SSNHL at age 40. We add to a body of research investigating the potential long-term impacts of auditory hallucinations on cognitive and sensory function and propose that auditory hallucinations may be a risk factor for SSNHL due to the impact of auditory hallucinations on various areas of the brain that are involved in auditory processing. This association could provide valuable insights for healthcare providers in managing and potentially preventing SSNHL. Future studies should further investigate the possible bidirectional relationship between auditory hallucinations and SSNHL with a larger sample size, taking into consideration the pathogenesis and long-term impacts of auditory hallucinations, as well as possible psychological/neurologic risk factors behind SSNHL.
